# Study on the Recognition of Coal Miners’ Unsafe Behavior and Status in the Hoist Cage Based on Machine Vision

**DOI:** 10.3390/s23218794

**Published:** 2023-10-28

**Authors:** Wei Yao, Aiming Wang, Yifan Nie, Zhengyan Lv, Shuai Nie, Congwei Huang, Zhenyu Liu

**Affiliations:** 1 School of Mechanical and Electrical Engineering, China University of Mining & Technology, Beijing 100083, China; 13705558@chnenergy.com.cn (W.Y.); 18855175309@163.com (Y.N.); 15238132238@163.com (Z.L.); nieshuaiv@163.com (S.N.); 15365430468@163.com (C.H.); 18810246174@163.com (Z.L.); 2 Digital Inteltech, CHN Energy, Beijing 100011, China

**Keywords:** coal mine hoist cage, visual monitoring method, Yolov5s, unsafe behavior

## Abstract

The hoist cage is used to lift miners in a coal mine’s auxiliary shaft. Monitoring miners’ unsafe behaviors and their status in the hoist cage is crucial to production safety in coal mines. In this study, a visual detection model is proposed to estimate the number and categories of miners, and to identify whether the miners are wearing helmets and whether they have fallen in the hoist cage. A dataset with eight categories of miners’ statuses in hoist cages was developed for training and validating the model. Using the dataset, the classical models were trained for comparison, from which the YOLOv5s model was selected to be the basic model. Due to small-sized targets, poor lighting conditions, and coal dust and shelter, the detection accuracy of the Yolov5s model was only 89.2%. To obtain better detection accuracy, k-means++ clustering algorithm, a BiFPN-based feature fusion network, the convolutional block attention module (CBAM), and a CIoU loss function were proposed to improve the YOLOv5s model, and an attentional multi-scale cascaded feature fusion-based YOLOv5s model (AMCFF-YOLOv5s) was subsequently developed. The training results on the self-built dataset indicate that its detection accuracy increased to 97.6%. Moreover, the AMCFF-YOLOv5s model was proven to be robust to noise and light.

## 1. Introduction

### 1.1. Background and Formulation of the Problem

The hoist cage is the sole means of transporting miners up and down an auxiliary shaft in coal mines. It is an integral part of coal mine safety management. According to coal mine safety regulations, miners are required to wear safety helmets when they enter underground coal mines. The safety management measures for miners entering and exiting coal mines stipulates that it is necessary to ascertain the number and categories of miners entering and exiting mines. Due to the lack of effective monitoring methods, miners riding the hoist cage without wearing safety helmets occurs. Furthermore, due to miners experiencing physical discomfort, the occurrence of miners suddenly fainting and falling down is difficult to detect in a timely manner. These situations pose hidden dangers to coal mine production safety [1].

Therefore, detecting miners’ unsafe behaviors (wearing or not wearing safety helmets) and their status (quantity and categories and falling down in the cage) in the hoist cage is of great significance to detecting sudden accidents in time, and ensuring coal mine production safety. However, due to the lack of effective object detection methods, manual observations are required for detecting whether miners in the cage are wearing safety helmets, and when they fall in the cage [2]. It is difficult to achieve timely and accurate detection via manual supervision. Hence, it is very important to implement rapid and precise monitoring of coal miners’ unsafe behaviors and their status in the hoist cage based on machine vision.

### 1.2. Literature Survey

Machine vision technology is used in security, transportation, building, and other industries. For instance, it can be used for the automatic inspection and tracking in crucial locations, for recognizing of vehicle licenses, fingerprints and human faces, and for online detection of industrial products. In the coal mining industry, to identify unsafe behaviors from the monitoring video, a Gaussian mixture model was used to extract moving targets, and a local binary pattern was applied to extract object features to develop the behavior model [3]. Irregular behavior was classified using the support vector machine (SVM) [4]. To detect and prevent miners from entering hazardous areas in an underground coal mine, the gray level co-occurrence matrix was adopted to describe textural features of the images, and a multi-feature fusion personnel detection algorithm was proposed based on the adaptive local similarity pattern [5]. Aiming at facial recognition of coal miners wearing helmets [6], a two-dimensional wavelet transformation-based Mallat algorithm was proposed for the image preprocessing and the generalized symmetry transformation-based algorithm was used to extract the image features, which solve the problem of identification accuracy being affected by contaminated face images caused by coal ash.

The above methods are the traditional image recognition-based methods that include three steps. Firstly, the target region is detected. Secondly, the features of the target region are extracted. Colors, textures, and shapes [7,8,9,10,11,12] are usually used to describe the feature of images, and the middle- or high-level semantic features are also utilized sometimes. Finally, the classifiers, such as the SVM model [12], naive Bayes model, and so on, are used for the recognition. The traditional image recognition-based methods can not accurately achieve multi-object detection. Therefore, they are not suitable for detecting the eight types of objects in this study. Moreover, the computation speeds of traditional methods are slow. In addition, the recognition accuracy is significantly affected by the harsh environment of coal mine hoist cages.

As a result, deep learning-based detection methods are proposed for the analysis of online monitoring video data in coal mines. Currently, deep convolutional neural networks are widely used for feature extraction, object classification, and location prediction. In addressing different working environments in coal mines, a deep neural network, which is composed of four convolution layers, three pooling layers, and three fully connected layers, was designed to identify whether miners were wearing their safety helmets [13]. To simultaneously identify miners not wearing helmets and illegal pedestrian behavior while driving, a lightweight convolutional neural network (CNN) model was proposed, using deep separable convolution and residual structure [14]. In order to identify coal mine miners’ irregular operations [15], an improved two-stream method was proposed, and a quantum genetics-based optimization algorithm was presented to address the problem of model overfitting. Although this approach’s detection accuracy is good, its speed is slow. Therefore, regression-based one-stage detectors represented by the You Only Look Once (YOLO) series [16,17,18,19,20,21,22,23] and single shot multi-box detector (SSD) [24], known for their high detection accuracy and speed, were used to identify coal mine miners’ unsafe behaviors. In order to identify whether security equipment is being worn, the SSD model was improved by replacing the feature extraction network visual geometry group (VGG-16) with MobileNet [25]. Based on the YOLOv3 model, miners’ irregular behaviors and environmental signs in underground coal mines could be identified [26]. The adaptability of deep learning-based recognition methods is good; they can automatically extract object features and realize multi-object detection. However, due to the presence of densely packed objects, multi-scale targets, including many small targets, coal dust, shelter, and poor lighting conditions in the captured images within the hoist cage, significant challenges exist in detecting unsafe behaviors and miners’ status inside the cage based on the deep learning-based recognition methods.

### 1.3. Scope and Contribution of This Study

In this study, to address the above difficulties, an attentional multi-scale cascaded feature fusion-based visual detection model (AMCFF) was developed to detect miners’ unsafe behaviors and status in the hoist cage. By means of collecting coal mine online monitoring video data, laboratory simulations, web crawler data, and data expansion, a dataset that includes images of workers wearing and not wearing helmets, images of fallen miners, images of miners wearing helmets with six different colors (red, black, white, blue, green, and yellow) was constructed for the training and validation of the proposed model. Using the dataset, a comparison among the typical detection models was performed to select the YOLOv5s model as the basic model. A BiFPN-based feature fusion network was proposed to address the issue of recognizing small and medium-sized targets. Given the difficulty of handling targets of interest in complex scenes with densely packed objects, a backbone network based on convolution and the CBAM (convolutional block attention mechanism) was designed to effectively extract features from complex images. To accurately detect the target, an attention detection head was introduced, and a bounding box regression calculation method based on the CIoU (complete intersection over union) loss function was proposed. The training results on the dataset collected by the visual monitoring system in the hoist cage indicate a detection accuracy of 97.6%. Moreover, the proposed model has been shown to exhibit robustness.

### 1.4. Organization of the Paper

This study is organized as follows. Section 1 provides an introduction to this topic and illustrates the existing literature. Section 2 presents the construction of the dataset for training the proposed AMCFF-YOLOv5s model. Section 3 presents the details of the model. The experimental validation is shown in Section 4, followed by key conclusions in Section 5.

## 2. Dataset Construction

To train and validate the proposed model, the dataset was collected through sampling coal mine online monitoring videos, laboratory simulations, and web crawlers. Coal mine miners should wear safety helmets with different colors according to their categories. Therefore, the number and categories of miners can be estimated based on the color and quantity of helmets that the miners in the hoist cage are wearing. Since the original images could not be used for training and validating the proposed model, eight object categories that included miners not wearing helmets, fallen miners, and miners wearing helmets with various colors (red, black, white, blue, green, and yellow) were marked manually in the database using LabellmageV1.2 software. According to Equation (1), the obtained XML format labels must be converted to TXT format to meet the requirements of training the proposed model.

(1) $$\{\begin{array}{c} x_{center}=\frac{x_{\min}+x_{\max}}{2\times W}\\ y_{center}=\frac{y_{\min}+y_{\max}}{2\times H}\\ w=\frac{x_{\max}-x_{\min}}{W}\\ h=\frac{y_{\max}-y_{\min}}{H} \end{array}$$

where $\left(x_{\min},y_{\min}\right)$ is the coordinate of the upper left point of the original annotation box; $\left(x_{\max},y_{\max}\right)$ is the coordinate of the low right point of the original annotation box; $\left(x_{center},y_{center}\right)$ is the coordinate of the center point of the converted annotation box; w is the left or right offset; h is the upper or lower offset; W represents the width of the image; and H represents the height of the image.

Overfitting occurs when the model is trained with a small dataset. However, it is difficult to enrich the dataset by manually changing the environment in the hoist cage to obtain different images. Furthermore, in actual cage monitoring videos, occurrences where miners are not wearing safety helmets and falling are infrequent. Therefore, obtaining a sufficient number of relevant images directly from monitoring videos is not feasible. In this research, 1000 images related to coal miners wearing safety helmets in various colors were obtained through web crawling, 1000 simulated images of fallen miners were generated under laboratory conditions, and 2000 images were extracted by sampling frames from online monitoring videos of miners going up and down underground coal mines using a hoist cage.

**Figure 1:**
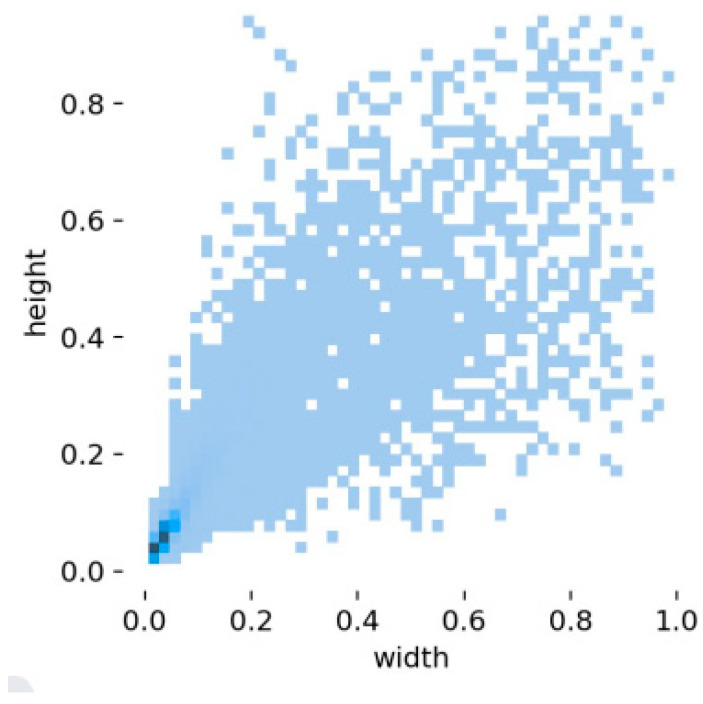
The statistical results of annotation boxes of each object category.

In the end, a total of 4000 original datasets were collected. Additionally, the original dataset was expanded to a total of 5000 images through data augmentation techniques such as image rotation, random image clipping, image scaling, adding Gaussian noise to images, and adjusting image brightness. The obtained dataset included 27,689 annotation boxes. Using an 8:2 ratio, the dataset was split into two parts: the training dataset and the validation dataset. Figure 1, which represents the statistical results of the size and distribution of the annotation boxes for each category, was generated by training the Yolov5 model using the dataset. According to Figure 1, in the lower-left corner, the target annotation boxes are most concentrated, and the targets in this area are small in size. In contrast, there are fewer target annotation boxes clustered in the upper right, lower right, and upper left corners, where the targets are larger in size. This indicates that there are more categories of small-sized objects in this dataset.

Small targets occupy a small area in the image, making their bounding box localization more challenging compared to larger or medium-sized targets. Additionally, there is limited visual information, making it difficult to extract discriminative features, and small targets are highly susceptible to environmental interference, posing a challenge for the detection model to accurately locate and identify them.

## 3. The Proposed AMCFF-YOLOv5s Model

### 3.1. The Classical Object Detection Models and Their Identification Results

Currently, in the field of object detection, deep learning methods can be primarily classified into two categories: two-stage object detection models and one-stage object detection models. The two-stage methods generate region proposals that may contain objects, and then classify and refine these proposals. These methods are relatively slower, as they require multiple runs of the detection and classification processes. On the other hand, one-stage object detection models are characterized by a one-step approach. They only need to feed the network once to predict all bounding boxes, making them relatively faster. Faster R-CNN is a typical representative of two-stage object detection models. SSD, YOLOv3, YOLOv3 + SPP, YOLOv3-Tiny, YOLOv5s, and YOLOv5m are typical representatives of one-stage object detection models. Therefore, the above seven models were compared by training based on the self-built dataset. The obtained detection accuracy and speed are shown in Table 1.

According to Table 1, the detection accuracies of YOLOv5m, YOLOv5s, and YOLOv3 + SPP are relatively high at 89.9%, 89.2%, and 89.5%, respectively. The detection speeds of SSD, YOLOv3-Tiny, and YOLOv5s are relatively high, with speeds of 86FPS, 62FPS, and 67FPS, respectively. Although the speed of SSD is the fastest, its accuracy is only 74.2%, while for YOLOv3-Tiny, its speed is slower than YOLOv5s and its detection accuracy is also lower than YOLOv5s. As for YOLOv5m and YOLOv3 + SPP, although their detection accuracy is a bit higher than YOLOv5s, their speeds are 13 FPS and 12 FPS slower than YOLOv5s, respectively. A high detection accuracy and high detection speed can simultaneously be achieved by YOLOv5s, with a detection accuracy of 89.2% and a speed of 67 FPS. Therefore, YOLOv5s was chosen as the basis for further improvements.

**Figure 2:**
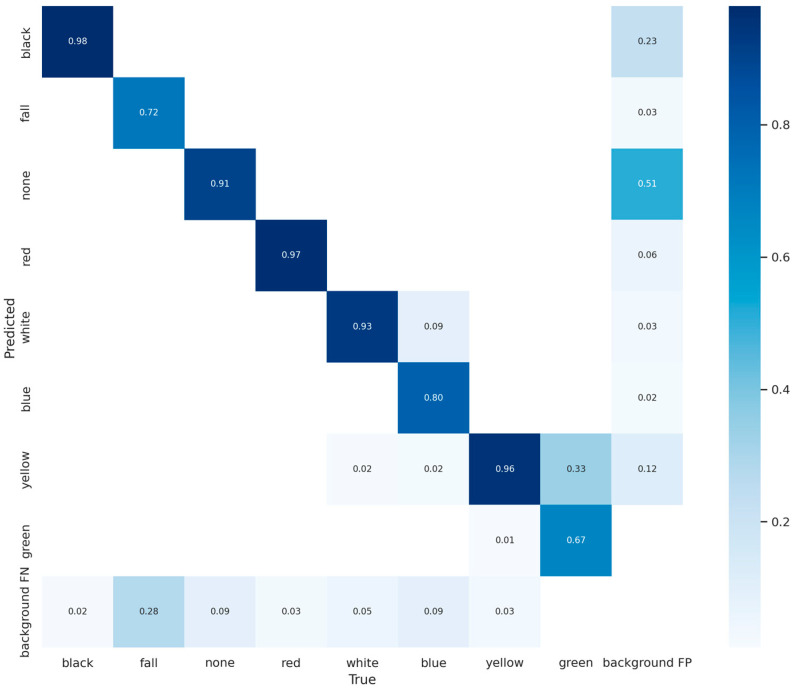
Confusion matrix of YOLOv5s. Illustration: background FP indicates objects that were not part of the background but were mistakenly identified as background, while background FN means objects that were part of the background but were incorrectly recognized as non-background.

**Figure 3:**
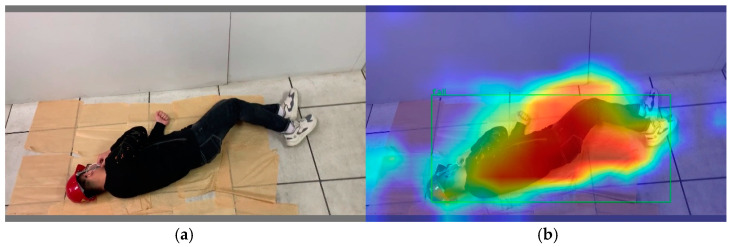
Classification heat map visualization for YOLOv5s: (**a**) the original image; (**b**) the classification heat map.

Figure 2 and Figure 3, which show the confusion matrix and classification heat map of the YOLOv5s model, were obtained using the dataset. According to Figure 2, the green helmet can be misclassified as other helmets, especially the yellow helmet, which has a false detection rate of 33%. Moreover, the fallen miner category is missed at a rate of 28%. According to Figure 3, the red region contributes significantly to object detection, while the blue region has a minimal contribution. It indicates that the detection accuracy and robustness of the YOLOv5s model for small-sized objects in the dataset are poor. Therefore, improvements are needed for YOLOv5s to achieve a high detection accuracy for identifying the unsafe behaviors and status of coal miners in the hoist cage.

### 3.2. The Proposed AMCFF-YOLOv5s Model

The proposed AMCFF-YOLOv5s model is composed of four main components: a backbone feature extraction network with the CBAM, attention detection heads that incorporate CBAM, an enhanced feature fusion network called WB-FFPN, and the CIoU responsible for loss computation. The backbone feature extraction network effectively and reliably extracts critical information from complex images, while WB-FFPN enhances the detection capability for small targets through multi-scale fusion. CIoU is used for loss computation to assist the model in accurately determining the true categories and contours of the targets.

#### 3.2.1. The Backbone Network and the Attention Detection Heads with CBAM

(1) CBAM Introduction

**Figure 4:**
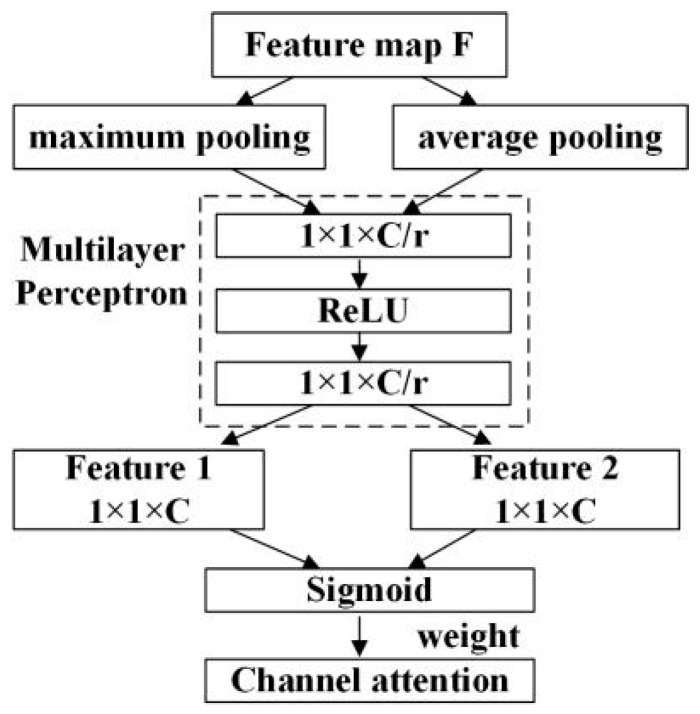
Channel attention module.

**Figure 5:**
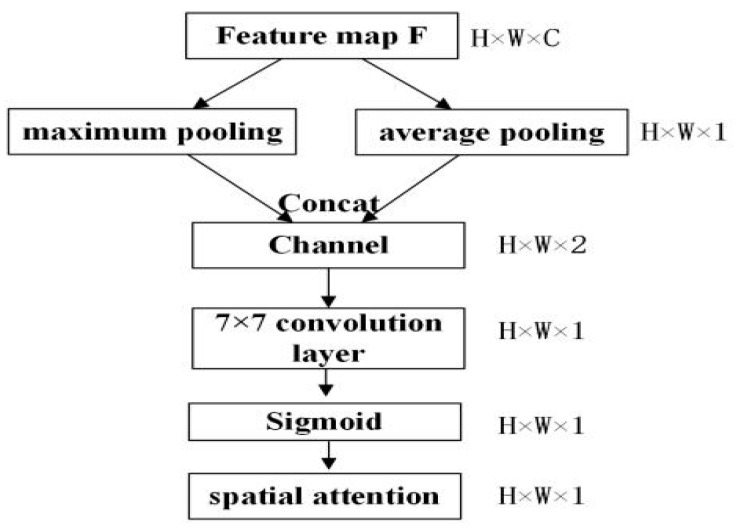
Spatial attention module.

The CBAM proposed by Woo [27] was applied in the backbone network and the attention detection heads, by which more attention is paid to the easily ignored objects. It is composed of the channel attention module shown in Figure 4, and the spatial attention module shown in Figure 5.

Figure 4 and Equation (2) represent the principle of the channel attention module. According to Figure 4, the input feature map of the channel attention module is H × W × C. Firstly, two 1 × 1 × C feature maps are obtained by processing the input feature map based on average pooling and maximum pooling. Secondly, the two obtained feature maps are subjected to the same convolution operation twice. The number of convolution kernels in the first convolution operation is C/r, and the activation function is ReLU. For the second convolution operation, the number of convolution kernels is C. Finally, the obtained feature maps are added together and the original input feature map is multiplied by a weight factor calculated by the sigmoid activation function to obtain the scaled feature map. Figure 5 and Equation (3) represent the principle of the spatial attention module. According to Figure 5, the input H × W × C feature map is initially processed by average pooling and maximum pooling to obtain the H × W × 1 feature maps. Secondly, they are concatenated, and then a 7 × 7 convolution layer is used to adjust the channel number. Finally, a weight factor calculated by the sigmoid activation function is used to multiply the original input feature map to obtain the scaled feature map.

**Figure 6:**
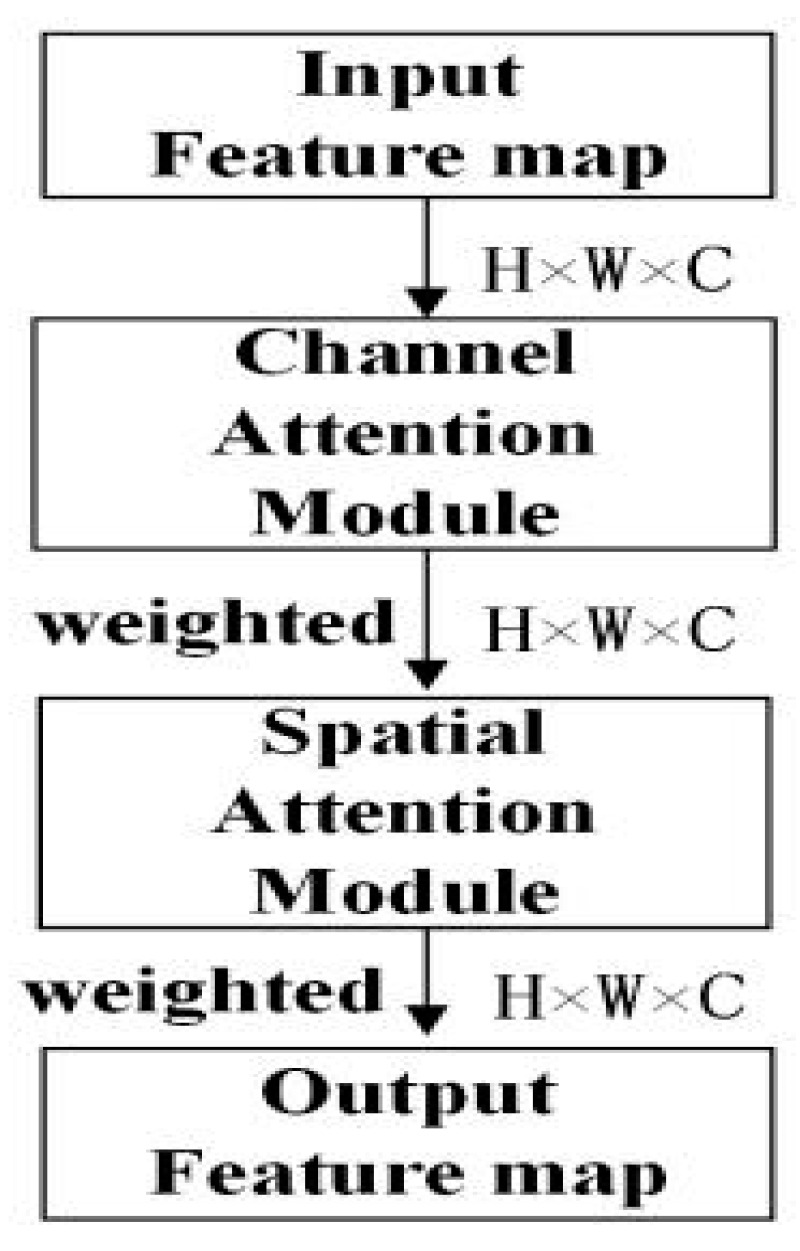
CBAM.

When using the channel attention module to learn the importance of each channel, it first compresses the spatial features of the feature map, and then learns in the channel dimension to obtain the importance of each channel. The spatial attention module can transform various types of data in space and automatically capture important area features. It can ensure that images, after undergoing operations such as cropping, translation, or rotation, still produce the same results as the original image before the operation. The CBAM shown in Figure 6 combines the channel attention module and the spatial attention module together. It first processes the input feature map with a channel attention module; the results are then processed through a spatial attention module, and finally, adjusted features are obtained. The CBAM can learn key points of objects in training, improve the importance of the target in the complex background, and increase the accuracy of object detection.

(2) $$\begin{array}{ll} M_{c}\left(F\right) & =\sigma \left(MLP\left(AvgPool\left(F\right)\right)+MLP\left(MaxPool\left(F\right)\right)\right)\\ & =\sigma \left(W_{1}\left(W_{0}\left(F_{avg}^{C}\right)\right)+W_{1}\left(W_{0}\left(F_{\max}^{C}\right)\right)\right) \end{array}$$

where F denotes the input feature map; H×W is the size of the feature map; C represents the number of channels; $AvgPool\left(F\right)$ is the average pooling operation of the feature map; $MaxPool\left(F\right)$ is the maximum pooling operation of the feature map; $F_{avg}^{C}$ is the global average pooling; $F_{max}^{C}$ is the global maximum pooling; $\sigma \left(\right)$ is the sigmoid function; $MLP\left(\right)$ is the multi-layer perceptron which builds the shared network with one hidden layer; and $W_{0}$, $W_{1}$ are the MLP weights.

(3) $$\begin{array}{ll} M_{s}\left(F\right) & =\sigma \left(f^{7*7}\left(\left[AvgPool\left(F\right),MaxPool\left(F\right)\right]\right)\right)\\ & =\sigma \left(f^{7*7}\left(\left[F_{avg}^{s};F_{\max}^{s}\right]\right)\right) \end{array}$$

where $F_{avg}^{s}$ is the global average pooling; $F_{\max}^{s}$ is the global maximum pooling; and $f^{7*7}\left(\right)$ represents the 7 × 7 convolution operation.

(2) The backbone network

**Figure 7:**
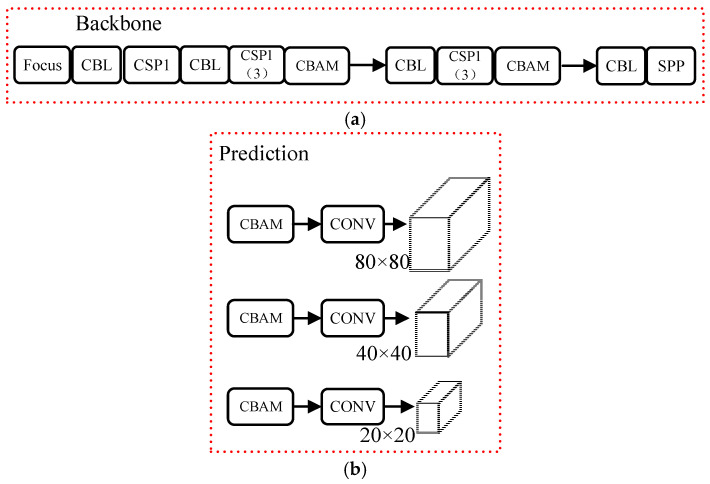
Three kinds of experiments: (**a**) add CBAM to the backbone network; (**b**) add CBAM to the detection head.

For convolutional neural networks, during the continuous convolution process, uniform convolution is applied to all features on the feature map, making it difficult to effectively highlight the key features of targets. In order to enhance the feature extraction capability for eight different types of targets within the region of interest while suppressing the influence of irrelevant information within the region of interest on the model’s feature weights, the CBAM is inserted into the middle and end sections of the DarkNet-53 network, as shown in Figure 7a. The CBAM is embedded into each CSP1 (3) module in the backbone network of YOLOv5s. With very few parameters, this enhances the feature extraction capability of the backbone network, thereby improving the initial feature extraction performance of the model. This allows the model to focus more on targets that may have been initially overlooked due to insufficient feature representation during the feature extraction process. As a result, it helps address the challenge of effectively extracting features from small and occluded targets within the region of interest.

(3) The attention detection head

In order to enable the model to more effectively recognize the objects of interest and address the issues of false positives and false negatives due to dim lighting conditions within the hoist cage, the CBAM is added to the detection head for detecting objects of different scales as shown in Figure 7b. The CBAM is added before each prediction convolution of YOLOV5s. This enhances the sensitivity of the detection head to key features, and improves the detection performance of the head for targets with insufficient feature representation, thus addressing the false positives and false negatives caused by low light conditions in the cage.

Studies were conducted for improving YOLOv5s using the CBAM according to Figure 7, and the training results are shown in Table 2. According to Table 2, the detection accuracy is improved by 4.6% and 1.9% compared with the original YOLOv5s, while the detection speed is only reduced by 1PFS when the CBAM is used. This indicates that the results can be improved by embedding the CBAM into the backbone network and the detection heads of YOLOv5s. The reason is that the improved backbone network pays more attention to the hidden and easily ignored targets, which improves the attention to small objects and features with different weight. Therefore, the object detection accuracy is increased.

#### 3.2.2. The Feature Fusion Network WB-FFPN

**Figure 8:**
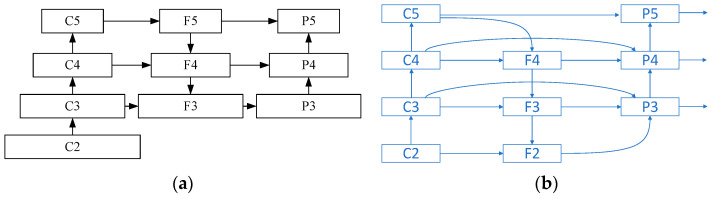
The feature fusion network: (**a**) original feature fusion network of YOLOv5s; (**b**) improved feature fusion network.

According to Figure 8a, in the original feature fusion network of YOLOv5s for features with different size, they are adjusted to the same resolution and are added using the same weight. However, different weights should be used because the contributions of features with different sizes are different. Therefore, a weighted and bidirectional feature fusion pyramid network (WB-FFPN) in Figure 8b was designed using the BIFPN and the strategy of concatenating the feature maps based on the channel. The fusion calculation formulas are as follows:

(4) $$F_{i}=Conv(Concat(C_{i},Resize(F_{i+1})))$$

(5) $$P_{i}=Conv\left(\frac{Concat\left(\omega_{1}\times P_{i},\omega_{2}\times F_{i},\omega_{3}\times Resize(P_{i-1})\right)}{\omega_{1}+\omega_{2}+\omega_{3}+\varepsilon }\right)$$

where $C_{i}$ is the multi-scale feature obtained by the feedforward; $P_{i}$ is the multi-scale feature for the detection head and $F_{i}$ is the intermediate multi-scale feature obtained in the top-down feature fusion; and $w_{1}$, $w_{2}$, $w_{3}$ are learnable feature fusion factors of $P_{i}$ and $F_{i}$ obtained in the down-sampling operation, and they are used to distinguish the importance of different features in the feature fusion process. Resize() represents the down-sampling or up-sampling operation; Concat() represents the concatenating operation; Conv() represents the convolution operation; ε=0.0001; i=1~5.

According to Figure 8 and Equations (4) and (5), the single input node F5 is deleted to simplify the network because it is not involved in the feature fusion. Moreover, the input and the output of each layer are directly connected to enrich the features. In the bottom-up path directly related to the output nodes, features with different scales from multiple input nodes are integrated to each node. In the integration, an additional weight, which is obtained by network learning according to their importance, is used for each input to achieve the effective feature fusion. Finally, features with three kinds of scales are obtained for object detection. The proposed feature fusion network can fully utilize the shallow features and integrate high-resolution features into the network to improve the detection accuracy of small targets.

#### 3.2.3. The Loss Function

The loss function is used to estimate the difference between the predicted value and the real value. It is crucial to improving the performance of the model. The loss function GIoU is utilized for YOLOv5s. It has a problem of convergence. The loss function CIoU shown in Equation (6) considers not only the overlap area, but also the centers’ distance and the aspect ratio. It converges faster, and its regression prediction bounding box is more accurate. Hence, the CIoU loss function was applied to replace the GIoU loss function in the proposed AMCFF-YOLOv5s model.

(6) $$\begin{array}{l} loss_{box}=1-CIoU=1-IoU+\frac{d^{2}}{c^{2}}+\alpha v\\ v=\frac{4}{\pi^{2}}{\left(\arctan\frac{w^{gt}}{h^{gt}}-\arctan\frac{w^{p}}{h^{p}}\right)}^{2}\\ \alpha =\frac{v}{\left(1-IoU\right)+\nu } \end{array}$$

where α is the weight; c is the distance between the center of the predicted bounding box and the center of the actual bounding box; d is the relative diagonal distance between the predicted bounding box and the actual bounding box; w and $w^{gt}$ are the width of the prediction bounding box and the width of the actual bounding box, respectively; and h and $h^{gt}$ are the height of the prediction box between the prediction bounding box and the height of the actual bounding box, respectively.

#### 3.2.4. Structure of AMCFF-YOLOv5s Model

**Figure 9:**
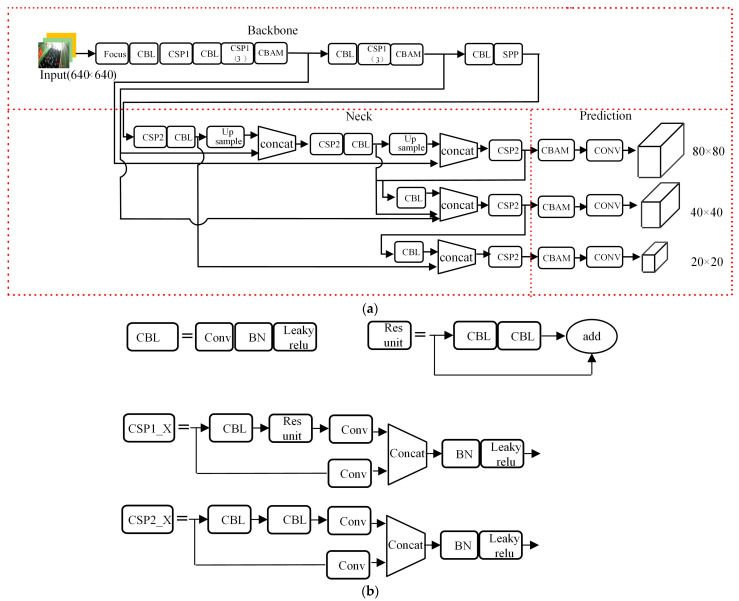
The structure of the proposed AMCFF-YOLOv5s model: (**a**) structure of AMCFF-YOLOv5s; (**b**) structure of CBL, Res unit, CSP1, and CSP2.

Figure 9 shows the structure of the proposed AMCFF-YOLOv5s model. It is composed of three parts, which are the input, the backbone network, the feature fusion network (neck), and the prediction. Based on the YOLOv5s model, to improve the detection accuracy of small objects and the robustness, the CBAM is added after the second and third down-sampling convolution modules in the backbone network; thus, more attention is paid to the easily ignored objects. Compared with YOLOv5s’ neck, which was developed based on feature pyramid networks (FPN) and path aggregation network (PAN) structure to realize multi-scale feature fusion, although high recognition accuracy can be obtained, the model parameters and the computational time of YOLOv5s are increased; these lead to low model efficiency. Moreover, according to the FPN and PAN structure, the feature maps are converted to the same size and are concatenated using the same weight, which cannot fully utilize the features with different scales. Using the different weights of features with different scales and the strategy of concatenating the feature maps based on the channel, the proposed WB-FFPN neck can improve the feature fusion efficiency and the detection accuracy of small-sized objects. To accurately detect the target, an attention detection head improved by CBAM was proposed.

As shown in Figure 9, batch normalization (BN), the leaky ReLU activation function, and residual units are extensively used in the CBL and CSP1 structures of the model’s backbone. Additionally, in the CBL and CSP2 structures of the neck, BN, and the leaky ReLU activation function are also employed. In theory, the leaky ReLU activation function remains constant at 1 in the positive region, thereby preventing the issues of vanishing and exploding gradients in deep networks. As for BN, it is a method that standardizes the output of each layer to have a consistent mean and variance, effectively eliminating the impact of weight parameter scaling and resolving the problems of gradient vanishing and exploding. The residual structure offers significant advantages in backpropagation, helping to mitigate gradient vanishing. Therefore, this model does not suffer from vanishing or exploding.

Moreover, the k-means++ clustering algorithm was proposed to calculate the prior bounding box, which is suitable for the self-built dataset. The prior bounding box, which was designed according to the self-built dataset, plays a critical role in improving the detection accuracy. There are nine groups of the prior bounding boxes in the YOLOv5s model. They are obtained based on the MS COCO dataset, which includes eighty object categories. For the self-built dataset, there are only eight object categories. Therefore, it was necessary to redesign the prior bounding boxes. To reduce computation time and classification errors, the k-means++ clustering algorithm was applied to calculate the prior bounding box using the following steps:

Step 1: Determine the number of categories, k, based on clustering, and randomly select a target point as the original cluster center of the self-built dataset.

Step 2: Calculate the matching probability P of the selected target points being the next cluster center. Determine the next cluster center, using P according to Equation (7):

(7) $$P=\frac{D{\left(x\right)}^{2}}{\sum_{x\in \lambda }D{\left(x\right)}^{2}}$$

where $D\left(x\right)$ represents the shortest distance between the target point of the self-built dataset and the existing cluster center.

Step 3: Calculate the IoU distance between the target point and the cluster centers; assign each target point to its nearest existing cluster center, and conduct the first iteration of all the target points.

Step 4: Calculate the cluster center again, and assign all the target points to the nearest cluster category until the cluster center remains unchanged.

According to the above steps, the average cross-merge ratio of the clustering results is calculated by gradually increasing k. When k is greater than 6, the average cross-merge ratio increases slowly. Therefore, the prior bounding boxes for the self-built dataset should be calculated when k equals 6. The obtained nine groups of prior bounding boxes are shown in Table 3. They are assigned to features with three scales for 8 times down-sampling, 16 times down-sampling, and 32 times down-sampling.

## 4. Experimental Study and Discussion

The proposed AMCFF-YOLOv5s model was trained on the self-built dataset for validation. Moreover, the detection results between the proposed model and YOLOv5s were compared. The ablation experiment was conducted to study the contribution of the k-means++ clustering algorithm, the proposed BiFPN-based feature fusion network, the CBAM, and the CIoU loss function to the proposed model. The detection results were affected by poor light conditions and coal dust in the hoist cage. Therefore, the robustness experiment was conducted by adjusting the brightness of the images in the self-built dataset, and by adding noise to the images.

To obtain the best performance for the proposed AMCFF-YOLOv5s model, the stochastic gradient descent method was utilized to optimize the loss function, and the attenuation weight was 0.0005. The learning rate warm-up method was applied, and the initial learning rate was 0.01. The cosine annealing strategy was used to adjust the learning rate when the model gradually stabilized. The resolution of the input images was 640 × 640. The online mosaic data augmentation method was used to ensure the robustness of the proposed network. To prevent the model from overfitting, the learning period was 300 epochs and the momentum was 0.937. The IoU threshold was 0.5, and the maximum number of training batches was 32. The training environment is summarized in Table 4.

### 4.1. Training Results and Discussion

**Figure 10:**
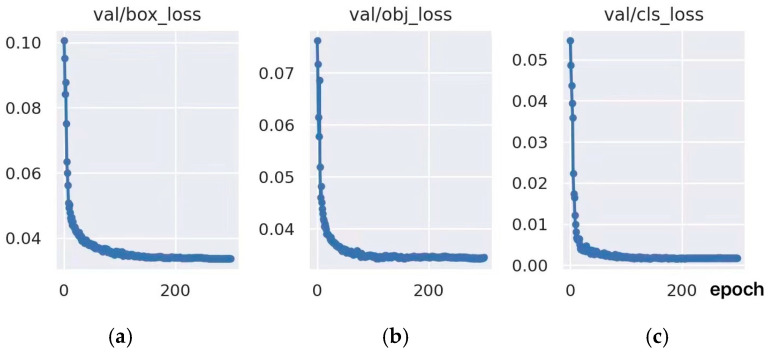
Curve of the loss function: (**a**) bounding box loss; (**b**) objectness loss; (**c**) confidence score loss.

Figure 10 shows the curve of the loss with the iterations number in the training process. According to Figure 10, the curve drops rapidly from 0 to 20 times iterations. After 200 times, the curve remains stable and the value almost equals zero. This indicates that the learning process is converging.

Table 5 and Table 6 represent the detection results of the proposed model, Yolov5s, and Yolov7. According to Table 5, compared with the Yolov5s model, the detection accuracy of the proposed model for wearing helmets with black color, red color, white color, yellow color, blue color, and green color increases by 0.6%, 3.4%, 5.1%, 4.1%, 11%, and 15.5% respectively. For the Yolov5s model, it is difficult to identify the blue and green helmets due to their similar color characteristics. However, for the proposed model, the detection accuracy of the blue and green helmets is greatly improved. Therefore, the average detection accuracy of the safety helmets with different colors increased from 92.13% to 98.75%. Moreover, the detection accuracies of not wearing helmets and fallen miners increased by 1.9% and 25.6%, respectively. Therefore, for the eight categories, the average detection accuracy significantly increased from 89.2% to 97.6%. This is because the focus of the proposed model on small targets is improved, which solves the problem of the poor detection accuracy of the Yolov5s model for small objects.

According to Table 1 and Table 5, compared with the commonly used methods Faster-RCNN, SSD, YOLOv3, YOLOv3-Tiny, YOLOv3 + SPP, and YOLOv5m, the average detection accuracies of the proposed model increased by 26.3%, 23.4%, 11.1%, 10.8%, 8.1% and 7.7%, respectively. According to Table 1, the best detection accuracies of single categories among the results obtained by the above methods are 92.6% for ‘not wearing a helmet’; 74.8% for ‘fallen miners’; 97.3% for ‘wearing black helmets’; 94.9% for ‘wearing red helmets’; 95.9% for ‘wearing white helmets’; 96.4% for ‘yellow helmets’; 97.3% for ‘wearing blue helmets’; and 99.5% for ‘wearing green helmets’. The proposed model only has a slight disadvantage in its detection accuracy of the ‘blue helmet’, being 1.7% lower than the 97.3% obtained by the YOLOv3-Tiny model. For the other seven categories, the detection accuracy of the proposed model is higher than those of the commonly used models. Especially for the ‘fallen miner’ category, the detection accuracy increases by 20.8%. When compared with YOLOv7, the average detection accuracy of the proposed model is a bit higher than that of YOLOv7, which is 95.6%. According to Table 6, the differences between YOLOv7 and the proposed AMCFF-YOLOv5s in detecting the categories of ‘not wearing helmet’ and ‘wearing helmets with different colors’ are very small, while their difference in identifying the ‘fallen miner’ category is large. For Yolov7, it is 86%, while for AMCFF-YOLOv5s, it is 95.6%. This indicates that the detection accuracy of the proposed model is high.

According to Table 6, the size of the proposed model is 16.2 MB, which is only 1.8 MB more than Yolov5s and 58.7 MB smaller than Yolov7. The detection speed is 59 FPS, which is only 8FPS slower than Yolov5s and is 7 FPS faster than that of Yolov7. However, according to Table 1, the proposed model is 6FPS, 4FPS, and 5FPS faster than that of YOLOv3, YOLOv3 + SPP, and YOLOv5m, respectively. This indicates that the detection speed of the proposed model is high. The proposed method meets the requirements for online monitoring of the status of coal mine miners in a hoist cage.

**Figure 11:**
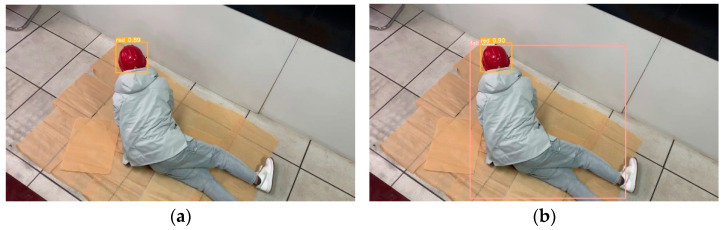
Simulation in the laboratory with a fallen person in a red helmet: (**a**) detection results of YOLOv5s; (**b**) detection results of the proposed model.

**Figure 12:**
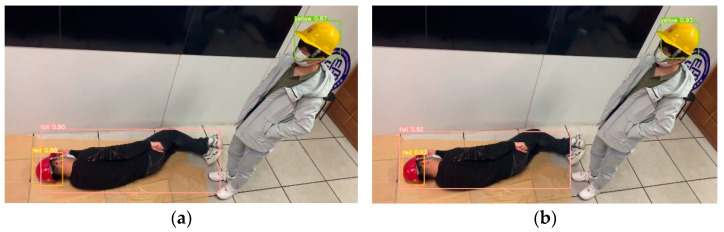
Simulation in the laboratory with a fallen person and a standing person in a red helmet and a yellow helmet, respectively: (**a**) detection results of YOLOv5s; (**b**) detection results of the proposed model.

**Figure 13:**
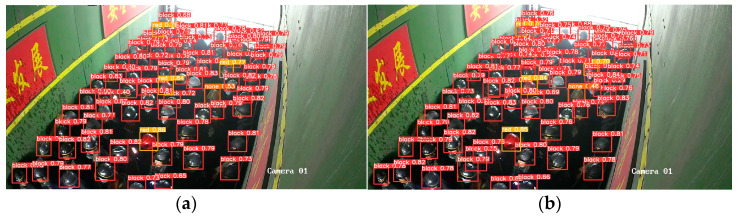
Multiple small objects in a coal mine hoist cage: (**a**) detection results of YOLOv5s; (**b**) detection results of the proposed model.

**Figure 14:**
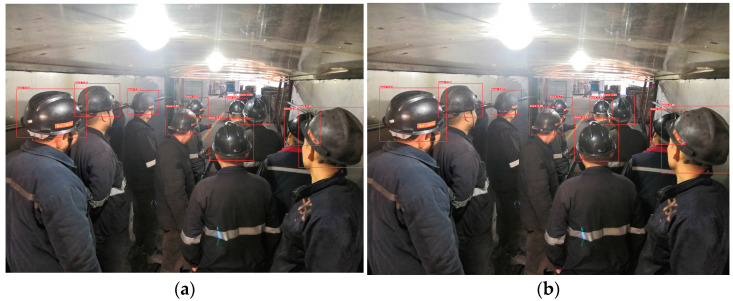
Multiple objects with different sizes in a coal mine hoist cage: (**a**) detection results of YOLOv5s; (**b**) detection results of the proposed model.

**Figure 15:**
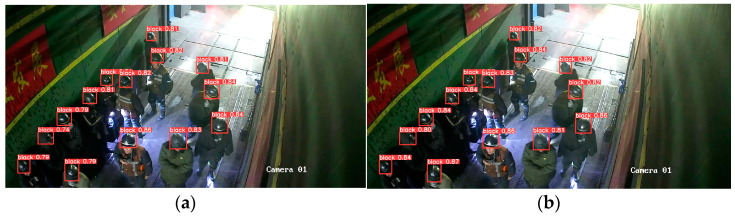
Multiple objects in a dim hoist cage with uneven light: (**a**) detection results of YOLOv5s; (**b**) detection results of the proposed model.

Figure 11, Figure 12, Figure 13, Figure 14 and Figure 15 show the visual results obtained by the proposed model and the YOLOv5s model, based on the test dataset. According to Figure 11, the fallen miner is marked with a pink box, and the red helmet is marked with an orange box. Using the YOLOv5s model, the red helmet is detected, but the fallen miner is missed. Meanwhile, for the proposed model, the two categories are both detected accurately. According to Figure 12, a standing miner is added into the image. The three categories, which are the fallen miner, the red helmet, and the yellow helmet, are all detected by the proposed model and the YOLOv5s model. However, the detection accuracy of the proposed model is higher. According to Figure 13, the helmets are small objects in the hoist cage, and the mutual occlusion phenomenon occurs. Therefore, some helmets are missed when using YOLOv5s. According to Figure 14, in the coal mine hoist cage, the close target is relatively large and the long-distance object is relatively small. All of the targets are detected using the two models, but the detection accuracy of the proposed model is higher. According to Figure 15, in a dim hoist cage with uneven light, the targets are small and fuzzy. All of the objects are detected; however, the detection accuracy of the proposed model is higher than that of YOLOv5s. From the above, the detection results of the proposed model are good, even though the conditions in the coal mine hoist cage are poor.

### 4.2. Ablation Experiment Results and Discussion

According to Table 7, the experiment was conducted by adding the k-means++ clustering algorithm, the proposed BiFPN-based feature fusion network, the CBAM, and the CIoU loss function to YOLOv5s to study the influence of each improvement measure. The experimental results are shown in Table 8.

According to Table 8, the average detection accuracy of YOLOv5s is 89.2%. When replacing GIoU loss function with CIoU loss function in Yolov5s, the detection accuracy increases by 1.7% and the detection speed is unchanged. This is because the CIoU loss function considers the center distance and the aspect ratio of the bounding box; thus, its convergence rate is faster, and it pays more attention to the central information of the bounding box. When utilizing the k-means++ algorithm to replace the k-means algorithm in Yolov5s, the detection accuracy increases by 1.1%, and the detection speed remains unchanged. Adding the CBAM to the backbone network of YOLOv5s has little effect on the detection speed, but the average detection accuracy is greatly improved by 2.7%. Especially for the fallen miner category, its detection accuracy increases 6.8%. The reason is that the introduction of the CBAM makes the model pay more attention to important features and focus on the target, while ignoring the effects of the background. Moreover, the number of model parameters is reduced. For the proposed BiFPN-based feature fusion network, the detection accuracy is improved by 2.9% and the detection speed, which is reduced by 7FPS, is also affected.

**Figure 16:**
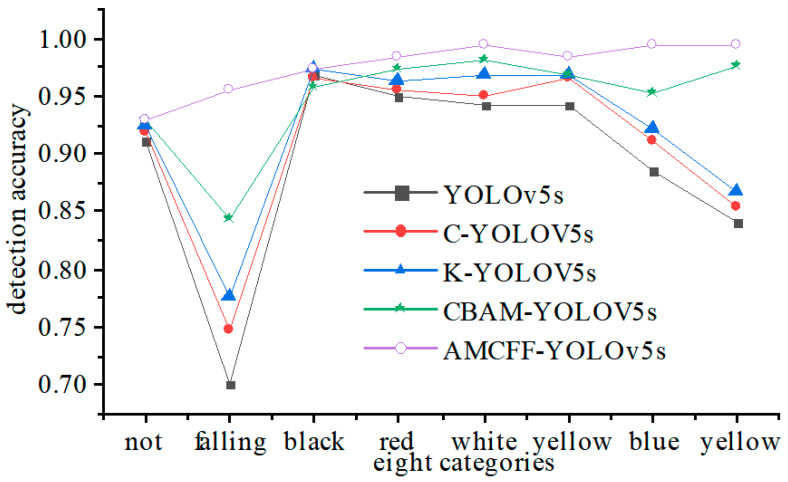
The effects of four improvement measures on the detection accuracy of each category.

Figure 16 reflects the effect of the four improving measurements on the detection accuracy of each category. According to Figure 16, the four measurements have a positive effect on the detection accuracy. For the black helmet category, although the CIoU loss function and the CBAM reduce the detection accuracy, the calculated bounding box and the proposed BiFPN-based feature fusion network have a positive influence on the detection accuracy, which is improved by 0.6%.

### 4.3. Robustness Experiment and Discussion

Poor lighting conditions and coal dust in the hoist cage can affect the detection accuracy of the proposed model. Therefore, 100 images were randomly selected from the self-built dataset, and their brightness was adjusted, and various types of noise were added to the images, as listed in Table 9. According to the results obtained in Table 9, the detection accuracy is reduced by 7.6% when 10% Gaussian noise is added to the images. When salt and pepper noise with a signal-to-noise ratio of 0.01 is introduced, the detection accuracy is reduced by 5%. With a signal-to-noise ratio of 0.01, the detection accuracy decreases by 6.3%. This is because salt and pepper noise and Gaussian noise blur the target features. Hence, feature extraction is affected. The higher the noise level, the less useful information the model can extract from the input. Hence, the low detection accuracy is obtained. As shown in Table 9, when the brightness is increased by 30%, the detection accuracy only decreases by 2.1%. When the brightness decreases by 30%, the detection accuracy only decreases by 3%. This indicates that the proposed model is robust to changes in the light brightness. The reason is that the online mosaic image enhancement algorithm is utilized in the model. Although image quality is impacted by lighting conditions and coal dust, and it reduces the detection accuracy, the accuracy of the proposed model is still above 90%. From the above findings, the proposed method is proven to be robust to noise and light brightness.

## 5. Conclusions

In order to estimate the number and categories of miners in a coal mine hoist cage, and to identify miners’ irregular behaviors such as not wearing safety helmets and falling down in the hoist cage, an object detection model called AMCFF-YOLOv5s was proposed. Experiments were conducted to validate the model. The main research and conclusions of this study are summarized as follows:

(1) A dataset, including images of miners wearing and not wearing helmets, fallen miners, miners wearing helmets with six different colors (red, black, white, blue, green, and yellow), was developed by means of sampling coal mine on-line monitoring video data, laboratory simulations, web crawler data, and data expansion such as image rotation, random cutting, and scale scaling. The commonly used detection models FAST-RCNN, SSD, YOLOv3, YOLOv3-Tiny, YOLOv3 + SPP, YOLOv5s, and YOLOv5m were studied based on the self-built dataset.
(2) The AMCFF-YOLOv5s model was proposed to solve the poor detection accuracy of small objects. The k-means++ clustering algorithm was utilized to calculate bounding boxes that are suitable for the self-built dataset. The WB-FFPN was introduced based on the BIFPN to replace the FPN + PAN structure. The CBAM was added into the backbone network of YOLOv5s. The CIoU loss function was applied to replace the GIoU loss function in YOLOv5s. By incorporating these four enhancements, the AMCFF-YOLOv5s model was developed. The training and validation results indicate that the detection accuracy of the proposed model is increased from 89.2% to 97.6%, and the detection speed is reduced from 69FPS to 58FPS. The detection accuracy for fallen miners improved from 70.1% to 95.6%. The proposed model meets the requirements for real-time and accurate detection of miners’ statuses in coal mine hoist cages.
(3) The proposed AMCFF-YOLOv5s model was compared with commonly used object detection models such as FAST-RCNN, SSD, YOLOv3, YOLOv3-Tiny, YOLOv3 + SPP, and YOLOv5m, based on the self-built dataset. Experimental results indicate that the detection accuracy of the proposed model is the highest. Using the ablation experiments, the proposed four improvement measures were shown to have positive influences on the detection accuracy. The results of the robustness experiments indicate that the light brightness has little influence on the detection accuracy. The impacts of Gaussian noise and salt and pepper noise on the detection accuracy are slightly more significant than that of the light brightness. However, the detection accuracy of the proposed model is still over 90%. This indicates that the AMCFF-YOLOv5s model is robust.

Only eight categories of miners’ statuses in coal mine hoist cages were investigated. For further studies, more categories should be explored. Poor image quality can lead to reduced detection accuracy; more image pre-processing algorithms should be employed. Moreover, to meet the requirements of field applications, the proposed model should be deployed to embedded devices. However, this may impact detection speeds. Therefore, model pruning methods should be explored.

## Figures and Tables

**Table 1:** Training results of the seven classical object detection models on the dataset.

Object Detection Model	Not Wearing Helmet	Fall	Black Helmet	Red Helmet	White Helmet	Yellow Helmet	Blue Helmet	Green Helmet	mAP	FPS
Faster-RCNN	0.408	0.491	0.861	0.771	0.761	0.808	0.833	0.77	0.713	20
SSD	0.622	0.584	0.788	0.7425	0.78	0.757	0.82	0.84	0.742	86
YOLOv3	0.575	0.748	0.926	0.944	0.942	0.955	0.914	0.915	0.865	53
YOLOv3-Tiny	0.602	0.630	0.921	0.925	0.934	0.964	0.973	0.995	0.868	62
YOLOv3 + SPP	0.912	0.705	0.963	0.944	0.959	0.938	0.922	0.817	0.895	55
YOLOv5s	0.91	0.701	0.968	0.949	0.943	0.943	0.885	0.84	0.892	67
YOLOv5m	0.926	0.734	0.973	0.942	0.952	0.935	0.893	0.835	0.899	54

**Table 2:** Training results of adding attention modules to YOLOv5s.

Experiments	Model Size (MB)	The Amount of Calculation (FLOPs)	Detection Speed (FPS)	Detection Accuracy (mAP)
YOLOv5s	14.4	15.9	67	0.892
Add CBAM to the backbone network	14.8	16.2	66	0.938
Add CBAM to the detection head	14.5	16.0	66	0.911

**Table 3:** The obtained prior bounding box based on k-means++ clustering algorithm.

Target Detection	Bounding Box
Small object detection	(11, 12) (20, 19) (28, 28)
Middle object detection	(43, 45) (67, 68) (111, 117)
Big object detection	(189, 190) (383, 155) (416, 332)

**Table 4:** The training environment.

Hardware		Software	
GPU	CPU	Develop environment	Operating system
Two NVIDIA GeForce GTX 3080	Intel(R) Xeon(R) 3204	PyTorch 1.10, Python 3.8.12	Ubuntu 18.04LTS

**Table 5:** Detection results of the eight categories of the proposed model, Yolov5s, and YOLOv7.

Model	Not Wearing Helmet	Fall	Black Helmet	Red Helmet	White Helmet	Yellow Helmet	Blue Helmet	Green Helmet
YOLOv5s model	0.91	0.701	0.968	0.949	0.943	0.943	0.885	0.840
YOLOv7 model	0.945	0.86	0.948	0.968	0.991	0.956	0.987	0.995
AMCFF-model	0.929	0.956	0.974	0.983	0.994	0.984	0.995	0.995

**Table 6:** Training results of the proposed model, Yolov5s, and YOLOv7.

Model	Size (MB)	Calculation (FLOPs)	Speed (FPS)	Accuracy (mAP)
YOLOv5s model	14.4	15.9	67	0.892
YOLOv7 model	74.9	103.3	52	0.956
AMCFF-model	16.2	17.3	59	0.976

**Table 7:** Design of ablation experiments. Illustration: the symbol ‘√’ represents the corresponding module is imported into YOLOv5s model.

	CIoU	k-Means++	CBAM	BiFPN-Based Feature Fusion Network
YOLOv5s				
C-YOLOv5s	√			
K-YOLOv5s	√	√		
CBAM-YOLOv5s	√	√	√	
AMCFF-YOLOv5s	√	√	√	√

**Table 8:** Detection results of eight categories in the ablation experiment.

Categories	YOLOv5s	C-YOLOv5s	K-YOLOv5s	CBAM-YOLOv5s	AMCFF-YOLOv5s
Not wearing a helmet	0.910	0.920	0.925	0.928	0.929
Falling workers	0.701	0.748	0.775	0.843	0.956
Wearing a black helmet	0.968	0.965	0.972	0.958	0.974
Wearing a red helmet	0.949	0.956	0.962	0.972	0.983
Wearing a white helmet	0.943	0.951	0.968	0.980	0.994
Wearing a yellow helmet	0.943	0.965	0.968	0.969	0.984
Wearing a blue helmet	0.885	0.912	0.922	0.952	0.995
Wearing a yellow helmet	0.84	0.855	0.867	0.975	0.995
Recall	0.97	0.97	0.97	0.98	0.99
Speed	67	67	67	66	59
Accuracy	0.892	0.909	0.920	0.947	0.976

**Table 9:** Results of robustness experiment.

Original Image	Gaussian Noise 10%	Salt and Pepper Noise 0.005	Salt and Pepper Noise 0.01	Brightness +30%	Brightness −30%
97.6%	90%	92.6%	91.3%	95.5%	94.6%

## Data Availability

The dataset is unavailable due to privacy.

## Abbreviations

CBAM	Convolutional block attention module
CIoU	Complete intersection over union
YOLO	You Only Look Once
YOLOv3	You Only Look Once version 3
YOLOv3-Tiny	You Only Look Once version 3 Tiny
YOLOv5s	You Only Look Once version 5s
YOLOv5m	You Only Look Once version 5m
AMCFF-YOLOv5s	Attentional multi-scale cascaded feature fusion-based YOLOv5s
SVM	Support vector machine
CNN	Convolutional neural network
BiFPN	Bidirectional feature pyramid network
SSD	Single shot multi-box detector
VGG	Visual geometry group
FAST-RCNN	Fast region-based convolutional neural network
SPP	Spatial pyramid pooling
mAP	Mean average precision
FPS	Frames per second
FPN	Feature pyramid network
PAN	Path aggregation network
WB-FFPN	Weighted and bidirectional feature fusion pyramid network
CA	Coordinate attention
MS COCO	Microsoft Common Objects in Context
IoU	Intersection over union
GIoU	Generalized intersection over union
MB	MByte
C-YOLOv5s	CIoU-improved YOLOv5s
K-YOLOv5s	k-means++-improved YOLOv5s
CBAM-YOLOv5s	CBAM-improved YOLOv5s
